# Assessment of renal congestion in a rat model with congestive heart failure using superb microvascular imaging

**DOI:** 10.1007/s10396-023-01396-7

**Published:** 2024-01-11

**Authors:** Tomofumi Nakatsukasa, Tomoko Ishizu, Ruriko Hayakawa, Masumi Ouchi, Naoto Kawamatsu, Kimi Sato, Masayoshi Yamamoto, Tomoko Machino-Ohtsuka, Kunio Kawanishi, Yoshihiro Seo

**Affiliations:** 1 Department of Cardiology, Institute of Medicine, University of Tsu Kuba, 1-1-1 Tennodai, Tsukuba, Ibaraki 305-8575 Japan; 2 Department of Medical Science, Institute of Medicine, University of Tsukuba, Tsukuba, Japan; 3 Department of Experimental Pathology, Institute of Medicine, University of Tsukuba, Tsukuba, Japan; 4 Department of Cardiology, Faculty of Medicine, Nagoya City University Graduate School of Medical Sciences, Nagoya, Japan

**Keywords:** Renal congestion, Congestive heart failure, Superb microvascular imaging

## Abstract

**Purpose:**

Renal congestion is a therapeutic target in congestive heart failure. However, its detailed evaluation in a clinical setting is challenging. This study sought to assess renal congestion impairment using superb microvascular imaging (SMI), a simple and accessible method.

**Methods:**

Dahl salt-sensitive rats, used as a model for congestive heart failure, underwent central venous pressure (CVP) measurements. Renal congestion was evaluated through measurements of renal medullary pressure (RMP) and assessment of renal perfusion using contrast-enhanced ultrasonography at both the early (control group) and heart failure phases (HF group). All rats were assessed with SMI. The region of interest (ROI) was set in interlobular vessels, interlobar vessels, and a combination of these areas. The area ratio was calculated from the color pixel count in the ROI divided by the total pixel count in the ROI. Intrarenal perfusion index (IRPI) was defined as (maximum area ratio—minimum area ratio) / maximum area ratio.

**Results:**

There were no significant differences in renal function and left ventricular ejection fraction between the two groups. CVP, time-to-peak (TTP) in the medulla, and RMP were higher in the HF group than in the control group. In the HF group, IRPI, evaluated in the interlobular vessels, was significantly higher than in the control group. IRPI was positively correlated with TTP in the medulla (p = 0.028, R = 0.60) and RMP (p < 0.001, R = 0.84), indicating that IRPI reflected renal congestion.

**Conclusions:**

IRPI is a useful tool for assessing renal congestion in rats with congestive heart failure.

**Supplementary Information:**

The online version contains supplementary material available at 10.1007/s10396-023-01396-7.

## Introduction

Elevated central venous pressure (CVP) is associated with impaired renal function and cardiovascular death [1–3]. The elevation of CVP correlates with renal congestion, defined as increased renal medullary pressure and impaired renal perfusion, both of which are poor prognostic factors for heart failure [4, 5]. Chronic renal congestion may result in renal dysfunction, including renal interstitial fibrosis, a known prognostic factor for heart failure in patients with congestive heart failure [6]. Consequently, renal congestion is a potential therapeutic target for congestive heart failure. Clinically, intrarenal venous flow patterns are used to assess renal congestion. However, this method only classifies the severity of renal congestion into three categories: continuous, biphasic, and monophasic. Animal studies have shown that even when renal medullary pressure is elevated and renal perfusion worsens, the intrarenal flow pattern remains the same—continuously. This finding suggests that the intrarenal flow pattern may not accurately reflect early or mild renal congestion [6, 7]. Superb microvascular imaging (SMI), which eliminates motion artifacts, enables high-frame-rate imaging and the evaluation of low blood flow velocities [8–10]. SMI can detect slower renal blood flow velocities than power Doppler ultrasonography, without the need for a contrast agent [11]. This study aimed to evaluate renal congestion in detail using SMI, a simple and accessible method.

## Materials and methods

Thirty-two male Dahl-Iwai S rats (DIS/Eis) (Japan SLC Inc. Shizuoka, Japan), which model hypertensive heart failure, were used. All rats were given a low-salt diet containing 0.3% NaCl until they reached the age of 6 weeks. Subsequently, they were transitioned to a high-salt diet containing 8% NaCl.

### Echocardiographic assessment

Transthoracic echocardiography (TTE) was performed concurrently with the SMI evaluation. All rats were given an inhalation anesthetic, isoflurane, with the dosage adjusted to maintain a heart rate between 300 and 350 beats per minute. This ensured they were adequately sedated for the invasive procedure. All echocardiographic images were obtained using a Vevo 2100 (VisualSonics Inc., Toronto, Canada) equipped with a 13- to 24-MHz linear transducer (MS-250). Left ventricular (LV) diastolic diameter (LVDd), systolic diameter, diastolic interventricular septum thickness (IVSTd), and diastolic posterior wall thickness (PWTd) were measured from a parasternal long-axis view. LV ejection fraction, end-diastolic volume (EDV), and end-systolic volume (ESV) were calculated using the Teichholz method.

### Hemodynamic studies

At the ages of 12, 18, and 21 weeks, blood pressure was measured using a tail-cuff system (CODA standard system, Hakubatec Life Science Solutions Co., Ltd., Tokyo, Japan). The rats were positioned on a heated surgical plate and anesthetized in the same manner as during the echocardiography procedure.

### Evaluation of renal congestion

Evaluation of renal artery and venous blood flow was performed using the same equipment used for echocardiography. The resistance index (RI) was measured as (maximum systolic blood flow velocity—end-diastolic blood flow velocity)/maximum systolic blood flow velocity [12]. The venous impedance index (VII) was calculated by dividing the difference between the maximum and minimum blood flow velocities of the renal interlobar veins by the maximum velocity [13].

The right femoral vein was exposed, and a 24-gauge indwelling catheter was inserted, to which a Y-connector was attached. A fiber optic pressure sensor (FISO-LS-PT9; FISO Technologies, Quebec, Canada) was inserted into the inferior vena cava to measure CVP. Subsequently, to measure renal medullary pressure (RMP), the left kidney was externalized, and a fiber optic pressure sensor was inserted into the renal medulla under echocardiographic guidance [6, 7, 14].

Contrast-enhanced ultrasonography (CEUS) was performed on the right kidney, which was not used for RMP measurements, using an Aplio device (Canon Medical Systems Co., Otawara, Japan) with a PLT-1204BT probe. This procedure quantified renal capillary circulation in the cortex and medulla. A perflubutane microbubble ultrasound contrast agent (Daiichi-Sankyo Co., Ltd., Tokyo, Japan) was administered as a 5-μL/kg bolus via the femoral vein, followed by a 5-mL saline injection [15]. Renal microcirculation was analyzed using the Vitrea Workstation (Canon Medical Systems Co., Otawara, Japan) [6, 7, 14]. Regions of interest (ROI) were identified in the cortex and medulla where the contrast enhancement was most uniform. A time-intensity curve was generated from the average signal intensity in dB within the ROI. In addition, time-to-peak (TTP, sec), defined as the time from the initial increase to peak intensity, was measured on the time-intensity curve.

### Superb microvascular imaging

**Fig. 1:**
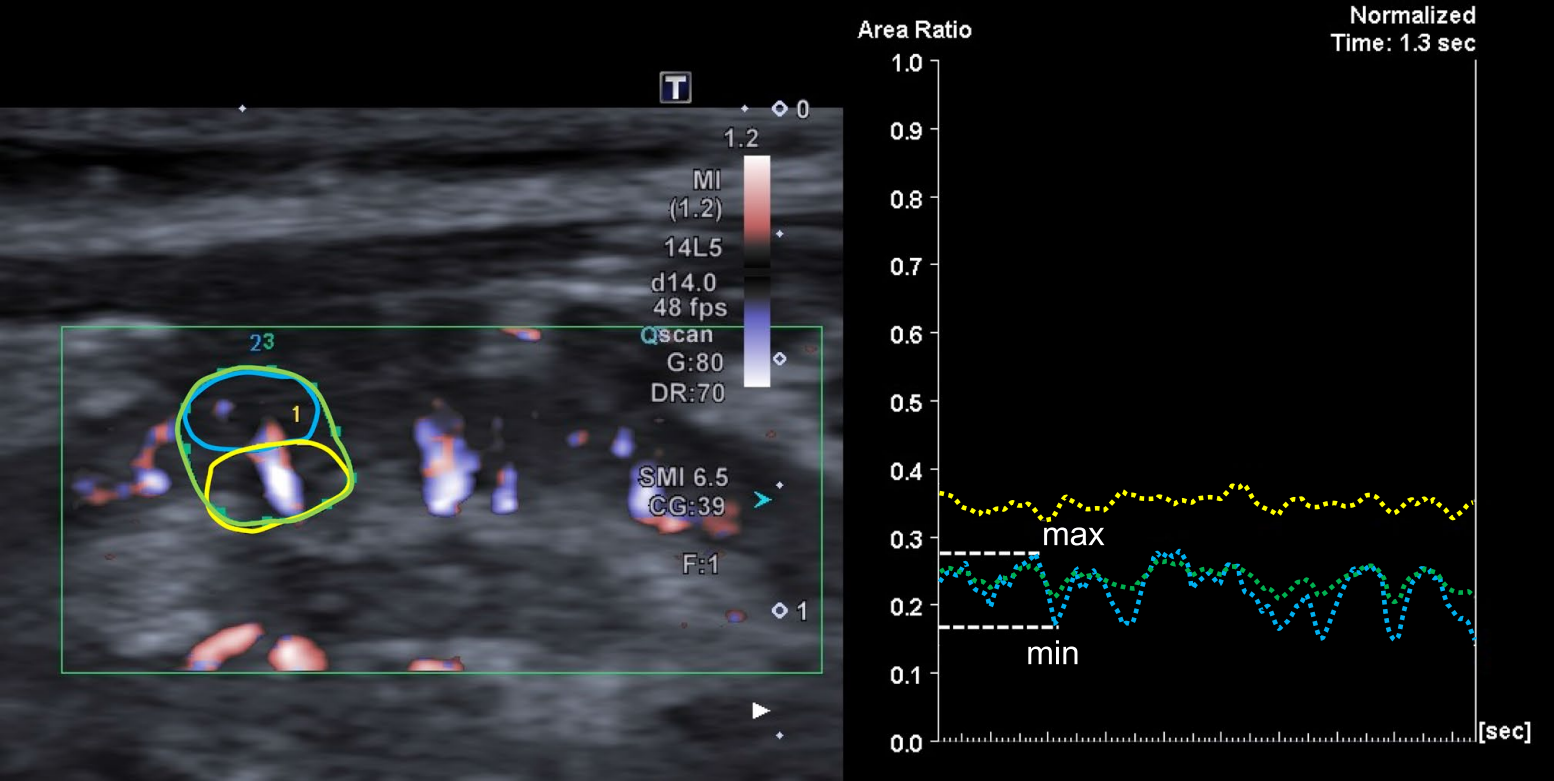
Measurement of IRPI. The left panel presents a long-axis view of the right kidney as visualized using SMI. The blue, yellow, and green circles respectively represent ROI for interlobular, interlobar, and both vessels. The right panel depicts a graph with the area ratio on the vertical axis and time on the horizontal axis. The area ratio, which is determined by dividing the number of blood flow pixels displayed on SMI by the number of pixels in the ROI, varies with pulsation. *ROI* region of interest, *SMI* superb microvascular imaging

SMI was performed using an Aplio (Canon Medical Systems Co., Otawara, Japan) with an attached probe. The right kidney, which underwent contrast-enhanced ultrasonography, was used for this evaluation. The ROI was designated in an interlobular artery and vein, an interlobar artery and vein, and in both artery and vein in images obtained from the right kidney. The SMI area ratio was calculated by dividing the number of pixels in the venous and arterial pulsatile ROIs by the total number of pixels in the ROI. Both the total number of pixels in the ROI and the number of pixels in the venous and arterial pulsatile ROIs were automatically calculated. The SMI area ratio was then obtained by dividing the latter by the former. The intrarenal perfusion index (IRPI) was subsequently determined as (maximum area ratio—minimum area ratio) / maximum area ratio (Fig. 1). These assessments were performed at the age of 12 weeks for early-stage heart failure (control group) and at 18 and 21 weeks for late-stage heart failure (HF group).

### Pathology

**Fig. 2:**
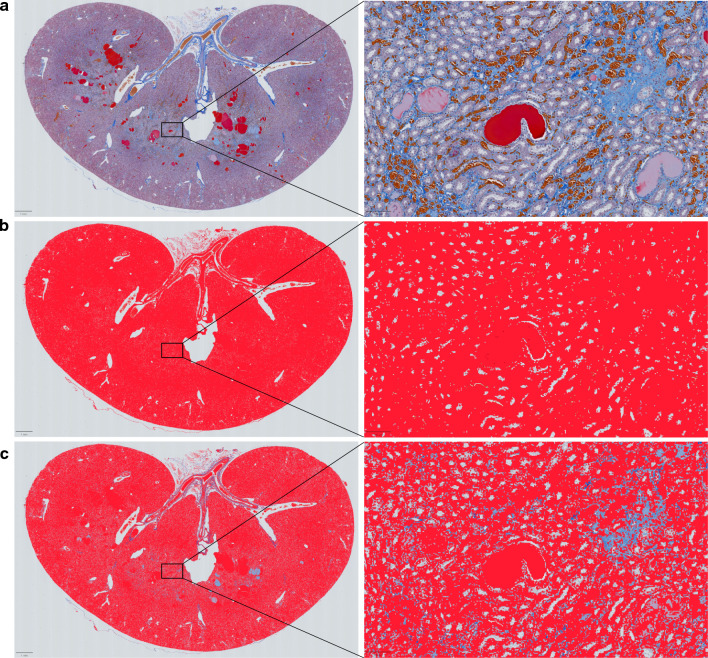
Renal interstitial edema. These panels show pathological images of Masson's trichrome-stained kidneys of a control subject. The left panel shows the right kidney stained with Masson's trichrome. The right panel is a higher magnification image of the boxed area in the left panel. Upper panels are raw Masson's trichrome staining (**a**). Middle panels show renal parenchyma in red using QuPath (**b**). The lower panels show the areas with edema in light blue (**c**)

The right kidney was fixed in 10% formaldehyde, embedded in paraffin, and sectioned into slices of 4-μm thickness. The specimens were stained with Masson's trichrome to evaluate renal edema. High-resolution imaging of the kidney tissue sections was performed using the NanoZoomer Digital Pathology System (2.0RS; Hamamatsu Photonics, Hamamatsu, Japan). Areas of renal edema, characterized by light blue stained tissue, were identified and their area ratios were measured using QuPath software (version 0.2.3) (Fig. 2) [16].

### Statistical analysis

The results of this study are presented as mean ± standard error. Rats aged 18 and 21 weeks were treated as a single group for analysis due to the similarity in their parameters. Organ weight divided by tibia length was used for analysis. The Shapiro–Wilk normality test was used to assess normal distribution. Owing to the non-normal distribution of CVP, RMP, and TTP in the medulla, natural logarithmic transformations were employed to meet the requirements for normality in the analysis. Differences between the control group and HF group were evaluated using the Mann–Whitney test. Correlations between parameters were assessed using Pearson’s correlation coefficient.

## Results

This study evaluated five, four, and five rats at the ages of 12, 18, and 21 weeks, respectively. All remaining rats died before evaluation. No significant difference was observed in terms of body weight between the two groups (Table 1). Notably, left ventricular weight and lung weight were significantly increased in the HF group. The latter was considered indicative of pulmonary edema and congestion. Kidney weights in the HF group were also significantly higher.

**Table 1:** Characteristics of hypertensive rats

	Control (n = 5)	HF (n = 9)	p value
Body weight	314.7 ± 16.4	344.4 ± 9.4	0.15
Organ weight			
Lung (mg/mm)	268.0 ± 15.3	489.7 ± 26.3	< 0.001
Left ventricle (mg/mm)	214.7 ± 6.2	309.9 ± 14.6	< 0.001
Kidney (mg/mm)	711.1 ± 24.4	793.1 ± 29.0	0.048
Hemodynamics			
sBP, mmHg	173.6 ± 18.5	201.8 ± 8.4	0.11
dBP, mmHg	123.2 ± 19.4	145.3 ± 8.5	0.35
HR,/min	414.2 ± 13.8	380.4 ± 27.9	0.44

*Mann–Whitney test. Values are the mean ± standard error

*dBP* diastolic blood pressure, *HR* heart rate, *sBP* systolic blood pressure

### Serum and urinary parameters

Table 2 presents the serum and urinary parameters. Renal function, assessed based on serum BUN and creatinine values, did not show any significant difference between the two groups. Moreover, creatinine clearance in the HF group was comparable to that in the control group. Urinary protein, albumin excretion, and L-FABP levels in the HF group did not exceed those in the control group.

**Table 2:** Serum and urinary data

	Control (n = 5)	HF (n = 9)	p value
Seum dara			
BUN, mg/dL	45.2 ± 2.9	36.1 ± 3.0	0.053
Cre, mg/dL	0.52 ± 0.06	0.62 ± 0.08	0.35
Urinary data			
TP, g/dL	299.8 ± 57.5	473.0 ± 69.6	0.078
Alb, g/dL	4370.8 ± 1720.7	4934.0 ± 614.6	0.77
Cre, mg/dL	24.6 ± 3.2	27.4 ± 4.3	0.61
L-FABP, ng/mg･Cr	9.1 ± 5.1	8.3 ± 1.0	0.88
CCR (mL/min)	1.86 ± 0.28	1.81 ± 0.19	0.89

*Mann–Whitney test. Values are the mean ± standard error

*Alb* albumin, *BUN* blood urea nitrogen, *CCR* creatinine clearance, *Cre* creatinine, *L-FABP* liver-type fatty acid-binding protein, *TP* total protein

Corrected L-FABP (ng/mgCr) equals low [?] L-FABP value (ng/ml) divided by urine creatinine (mg/dl) *100

### Ultrasonography

Echocardiographic parameters are shown in Table 3. Left ventricular hypertrophy, evaluated based on IVSTd and PWTd, was similar between the two groups. However, the HF group demonstrated significantly larger LVDd and LVEDV values compared to the control group. Despite these differences, LVEF was not lower. Renal ultrasound showed no significant differences in RI and VII between the two groups.

**Table 3:** Echocardiography, renal echo data

	Control (n = 5)	HF (n = 9)	p value
Echocardiography			
IVSTd (mm)	2.2 ± 0.1	2.1 ± 0.1	0.68
PWTd (mm)	2.4 ± 0.1	2.5 ± 0.1	0.83
LVDd (mm)	6.7 ± 0.1	7.7 ± 0.3	0.004
LVDs (mm)	4.7 ± 0.2	5.9 ± 0.4	0.02
LVEDV (mL)	230.6 ± 7.5	320.6 ± 24.9	0.006
LVESV (mL)	105.5 ± 10.6	178.5 ± 23.9	0.016
SV (mL)	125.1 ± 12.7	142.3 ± 21.7	0.5
LVEF (%)	54.0 ± 12.7	45.0 ± 20.1	0.28
Renal echo			
RI	0.61 ± 0.03	0.62 ± 0.01	0.83
VII	0.32 ± 0.06	0.27 ± 0.02	0.52
Organ weight			
Lung (mg/mm)	268.0 ± 15.3	489.7 ± 26.3	< 0.001
Left ventricle (mg/mm)	214.7 ± 6.2	309.9 ± 14.6	< 0.001
Kidney (mg/mm)	711.1 ± 24.4	793.1 ± 29.0	0.048

*Mann–Whitney test. Values are the mean ± standard error. All organ weight values are divided by the tibia length

*IVSTd* diastolic intraventricular septum thickness, *LVDd* left ventricular diastolic diameter, *LVDs* left ventricular systolic diameter, *LVEDV* left ventricular end-diastolic volume, *LVEF* left ventricular ejection fraction, *LVESV* left ventricular end-systolic volume, *PWTd* diastolic posterior wall thickness, *RI* renal resistance index, *SV* stroke volume, *VII* venous impedance index

### Hemodynamics and renal congestion

**Fig. 3:**
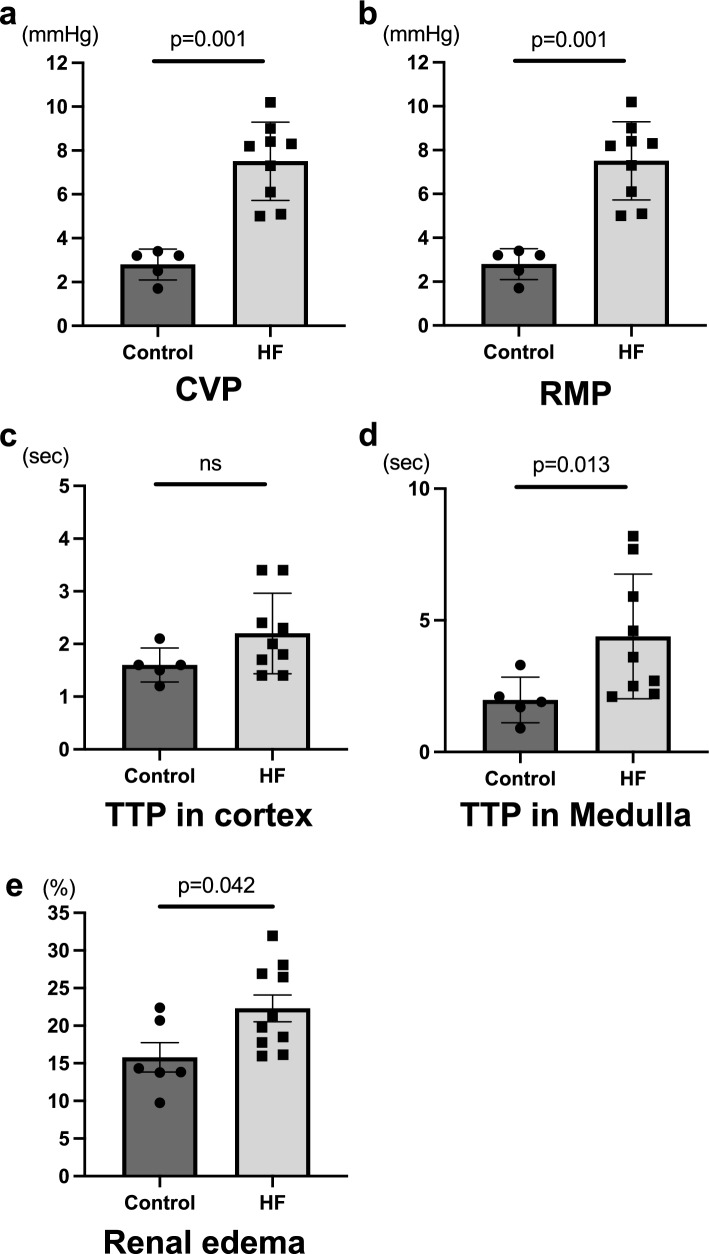
CVP and renal congestion. These panels show the central venous pressure (**a**) and renal congestion parameters RMP (**b**), TTP in cortex (**c**), TTP in medulla (**d**), and renal edema (**e**). Error bar means standard error. Differences between the two groups were evaluated using Mann–Whitney test. *CVP* central venous pressure, *HF* heart failure, *RMP* renal medullary pressure, *TTP* time to peak.

The HF group exhibited a significant increase in CVP (10.36 ± 1.54 mmHg vs. 18.34 ± 2.95 mmHg, p = 0.001) and RMP (2.80 ± 0.70 mmHg vs. 7.51 ± 1.79 mmHg, p = 0.001) compared to the control group (Fig. 3). TTP in the medulla was later in the HF group (1.98 ± 0.87 s vs. 4.39 ± 2.37 s, p = 0.013), while TTP in the cortex remained consistent between the two groups (1.60 ± 0.32 vs. 2.20 ± 0.76, p = 0.136). A strong correlation was observed between TTP in the medulla and RMP (R = 0.84, p < 0.001). Additionally, renal edema was significantly worse in the HF group compared to the control group (15.8 ± 1.9% vs. 22.3 ± 1.8%, p = 0.042) (Supplementary Fig. 1).

### IRPI

**Fig. 4:**
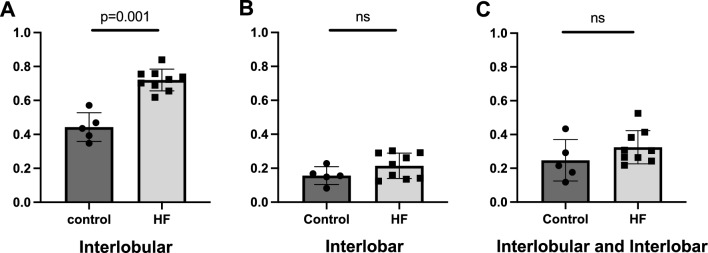
IRPI. A comparison of IRPI between control and HF groups is shown. The ROI was set in interlobular (**a**), interlobar (**b**) and interlobular and interlobar artery and vein (**c**). *HF* heart failure, *IRPI* intrarenal perfusion index, *ROI* region of interest, *SMI* superb microvascular imaging. Error bar means standard error. Differences between the two groups were evaluated using Mann–Whitney test. *HF* heart failure, *IRPI* intrarenal perfusion index, *ROI* region of interest, *SMI* superb microvascular imaging

A significant difference was observed in terms of IRPI evaluated in the interlobular artery and vein between the two groups (Fig. 4). In the HF group, it was higher than that in the control (0.443 ± 0.09 vs. 0.72 ± 0.06, p = 0.001). Conversely, no significant difference was observed in IRPI calculated in the interlobar artery and vein (0.16 ± 0.05 vs. 0.21 ± 0.08, p = 0.156) or in both of them (0.24 ± 0.54 vs. 0.32 ± 0.03, p = 0.189).

### Relationship between renal congestion evaluation and IRPI

**Fig. 5:**
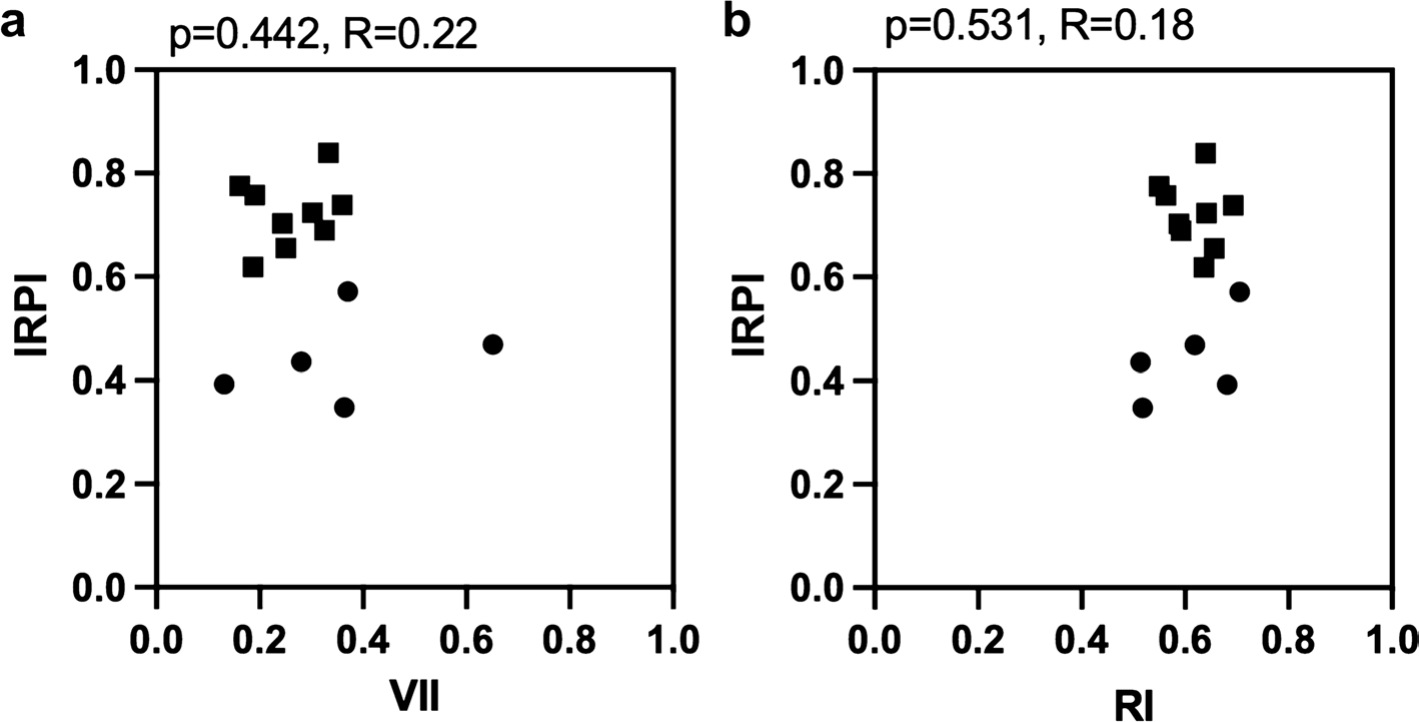
Correlation between pulse Doppler parameters and IRPI. These panels demonstrate the correlation between IRPI and pulse Doppler parameters, including VII (**a**) and RI (**b**). *IRPI* intrarenal perfusion index, *RI* superb microvascular index, *VII* venous impedance index

**Fig. 6:**
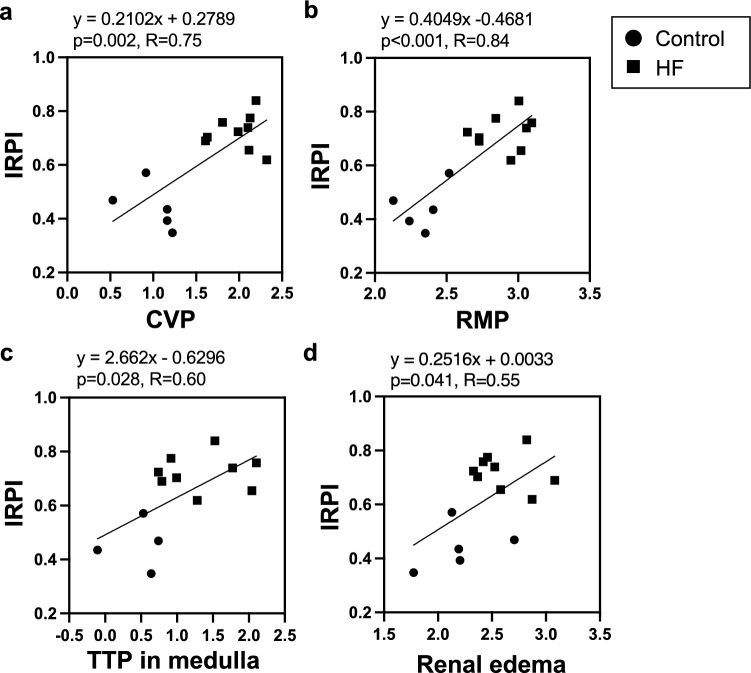
Relationship between IRPI and CVP, RMP, TTP in medulla, and renal edema. Owing to the non-normal distribution of CVP (**a**), RMP (**b**), TTP in the medulla (**c**), and renal edematous area (**d**), natural logarithmic transformations were employed to meet the requirements for normality in the analysis. *CVP* central venous pressure, *IRPI* intrarenal perfusion index, *RMP* renal medullary pressure, *TTP* time to peak

IRPI was not correlated with RI and VII (Fig. 5). Figure 6 illustrates the relationship between IRPI and CVP, as well as renal congestion indicators (RMP, TTP in the medulla, and renal edema). IRPI was found to correlate with CVP (p = 0.002, R = 0.75), RMP (p < 0.001, R = 0.84), medullary TTP (p = 0.028, R = 0.60), and renal edema (p = 0.041, R = 0.55). Therefore, IRPI appears to be influenced by both CVP and renal congestion.

## Discussion

SMI is a technique capable of depicting very slow blood flow at high frame rates, reducing clutter and eliminating tissue motion artifacts, all without the use of contrast agents. Our study indicates that IRPI, as assessed in the interlobular region, escalates with the progression of congestive heart failure. Furthermore, we found a positive correlation between the severity of renal congestion and IRPI, suggesting that the latter is influenced by renal congestion. To our knowledge, this is the first study to associate IRPI with renal congestion.

### IRPI in the interlobular region

In congestion due to obstructive uropathy, acute occlusion reduces common renal artery blood flow and increases venous blood flow velocity at the site of occlusion, resulting in decreased pulsatility of venous blood flow [17]. On the other hand, in congestive heart failure, interlobular arterial and venous pulsatility is elevated. Prior research has shown that the pattern of continuous interlobar venous flow transitions from biphasic to monophasic, depending on the severity of renal congestion [4, 5]. In addition, renal parenchymal pressure has been shown to increase. Therefore, in chronic heart failure, renal vein pulsatility has been shown to increase as congestion worsens. However, in our study, the interlobar vein flow pattern remained continuous in all rats, and furthermore, the venous impedance index (VII) did not differ between the control and HF groups. Consequently, more detailed assessment of renal congestion using conventional pulsed Doppler techniques proves challenging.

SMI technology is capable of detecting very slow velocities that may be overlooked on conventional pulsed-wave Doppler, and SMI can measure slight pulsations occurring in the renal blood flow [9]. Interestingly, our study found that, while blood flow pulsatility in the interlobar artery and vein as assessed using IRPI was not high in the HF group, there was significantly higher pulsatility in renal interlobular vessels. This suggests that renal interlobular blood flow pulsatility increases before renal congestion impacts the pulsatility of renal interlobar blood flow. In other words, IRPI may be able to identify early-stage renal congestion before the interlobar venous blood flow pattern changes from continuous to biphasic using pulsed Doppler methods. IRPI is indexed using area ratio. Area ratio is calculated by dividing the number of pixels drawn using SMI by the total number of pixels in the ROI. When evaluating raw pixel numbers, it affects the size of the ROI. Since IRPI is used to evaluate the rate of change, the rate of change does not change even if the ROI is large and contains more non-pulsating regions, as long as the blood vessels to be evaluated are included. On the other hand, IRPI, which is calculated using the number of blood flow pixels displayed within the ROI, can be influenced by highly pulsatile blood flow and even slight disruptions of blood flow during the cardiac cycle. Therefore, SMI may not be able to assess exacerbated renal congestion, and advanced renal congestion should be assessed based on interlobar venous flow patterns using pulsed Doppler.

### SMI assessment of renal congestion

Rats in the heart failure group not only had higher CVP but also exhibited decreased renal perfusion and increased renal interstitial pressure, changes that are typical in renal congestion. These changes, also observed in our study, are indicative of the phenotype of renal congestion: elevated CVP increases renal interstitial pressure and exacerbates renal perfusion. Furthermore, our renal pathological findings revealed edematous renal interstitium in the heart failure group, suggesting that elevated CVP in heart failure leads to fluid leakage into the renal interstitium. IRPI was positively correlated with exacerbation of renal congestion. The autoregulatory mechanism prioritizes preserving medullary renal perfusion, the most vulnerable part of the kidney, often at the expense of renal cortical perfusion [18, 19]. The renal interlobular arteries are resistance vessels. The juxtamedullary glomeruli supply the medulla with blood and initially branch off from the interlobular arteries. Therefore, it has less effect on vascular resistance than cortical perfusion and has less effect on medullary perfusion, which supplies less than 10% of total renal blood flow [20, 21]. In addition, renal medullary perfusion has less effect on vasoconstriction stimuli such as angiotensin II, noradrenaline, and renal nerve stimulation, which are activated by heart failure, than renal cortical perfusion [22]. Renal medullary perfusion assessed via CEUS was decreased, suggesting that cortical vascular pulsation increased as a result of sacrificing of cortical perfusion in order to maintain medullary perfusion.

Elevated IRPI may suggest a decrease in renal cortical perfusion and hemodynamic instability in the renal cortex caused by renal congestion, which could lead to worsened renal medullary perfusion. Consequently, renal interstitial pressure rises and renal perfusion decreases, leading to a decrease in the glomerular filtration rate. A decrease in estimated glomerular filtration rate (eGFR) exacerbates the long-term prognosis of heart failure [23–25]. In addition, venous stasis reduces urine volume, and renal congestion reduces renal medullary blood flow, which subsequently increases sodium retention in the renal tubules and exacerbates fluid overload in heart failure [26, 27]. Therefore, addressing renal congestion at an early stage of heart failure may lead to improvements in both renal and cardiac prognoses [28, 29]. Consequently, the mitigation of renal congestion may be a therapeutic target in heart failure management.

While CEUS can quantitatively evaluate renal congestion, it is an invasive method that requires the use of a contrast agent, such as Sonazoid® [30]. In this study, we found positive correlations between IRPI and CVP, RMP, TTP in the medulla, and renal edema. These results indicate that exacerbation of renal congestion influences IRPI. SMI, being a noninvasive method, can be used to assess renal congestion, and its improvement with treatments such as diuretics may enhance the prognosis. In other words, IRPI-guided medical therapy could prove beneficial as an initial evaluation method for renal congestion.

Our research highlights the potential of IRPI as a noninvasive indicator of renal congestion in heart failure. Its ability to detect early-stage renal congestion and its correlation with established renal congestion markers makes it a promising tool for managing heart failure and associated renal complications. Future research should further explore its clinical implications and utility in therapeutic strategies for heart failure.

### Study limitations

There are several limitations in this study. First, SMI does not differentiate between arteries and veins. Consequently, we could not determine whether the observed pulsatility in IRPI was arterial or venous. However, the positive correlation of IRPI with the renal congestion index in this study suggests that it is, at the very least, influenced by renal congestion.

Second, this study was an animal experiment, and all evaluations using IRPI were performed on rats. Due to the size differences in the vessels between humans and rats, it is unclear if these results can be directly applied to patients with heart failure in a clinical situation. In fact, the size of renal vessels in rats is much smaller than that in humans, limiting what SMI can visualize. With the potential to visualize a greater number of blood vessels in humans, it may be possible to evaluate the pulsatility of blood vessels in more detail. Future clinical studies are essential to confirm these findings in human subjects.

Third, although the difference was not statistically significant, the BUN/Cr ratio may have differed between the two groups, given that the HF group had slightly higher BUN and lower creatinine. Because the HF rats consumed a high-salt diet, they drank a lot of water, which may have been affected by hemodilution. In addition, it is conceivable that the condition gradually deteriorated in the later stages, and the amount of food intake decreased, resulting in insufficient protein intake. However, we did not measure actual food or water intake, so we are unable to provide results.

Lastly, we did not investigate whether improvements in renal congestion via the administration of diuretics would lead to improvements in IRPI. As a result, this study could not conclude whether the observed changes in IRPI were reversible or irreversible. Future studies, including therapeutic interventions, are required to resolve these issues.

## Conclusions

IRPI proved valuable for evaluating renal congestion in rats with congestive heart failure. Specifically, SMI was able to detect impairments in renal blood flow pulsatility prior to changes in the renal intravenous flow pattern as assessed by pulsed-wave Doppler. This suggests that IRPI-guided management of renal congestion could potentially enhance the prognosis of heart failure.

## Supplementary Information

Below is the link to the electronic supplementary material.

Supplementary file1 (DOCX 9044 KB)
