# Correction: Assessment of renal congestion in a rat model with congestive heart failure using superb microvascular imaging

**DOI:** 10.1007/s10396-025-01533-4

**Published:** 2025-03-28

**Authors:** Tomofumi Nakatsukasa, Tomoko Ishizu, Ruriko Hayakawa, Masumi Ouchi, Naoto Kawamatsu, Kimi Sato, Masayoshi Yamamoto, Tomoko Machino-Ohtsuka, Kunio Kawanishi, Yoshihiro Seo

**Affiliations:** 1 Department of Cardiology, Institute of Medicine, University of Tsu Kuba, 1-1-1 Tennodai, Tsukuba, Ibaraki 305-8575 Japan; 2 Department of Medical Science, Institute of Medicine, University of Tsukuba, Tsukuba, Japan; 3 Department of Experimental Pathology, Institute of Medicine, University of Tsukuba, Tsukuba, Japan; 4 Department of Cardiology, Faculty of Medicine, Nagoya City University Graduate School of Medical Sciences, Nagoya, Japan

**Correction: Journal of Medical Ultrasonics (2024) 51:159–168** 10.1007/s10396-023-01396-7

The article “Assessment of renal congestion in a rat model with congestive heart failure using superb microvascular imaging”, written by Tomofumi Nakatsukasa, Tomoko Ishizu, Ruriko Hayakawa, Masumi Ouchi, Naoto Kawamatsu, Kimi Sato, Masayoshi Yamamoto, Tomoko Machino‑Ohtsuka, Kunio Kawanishi and Yoshihiro Seo, was originally published under exclusive license to The Author(s), under exclusive licence to The Japan Society of Ultrasonics in Medicine. As a result of the subsequent decision to publish the article under the open access model, the article’s copyright notice was changed on 13 March 2025 to ©The Author(s) 2024 and the article is now distributed under a Creative Commons Attribution 4.0 International License, which permits use, sharing, adaptation, distribution and reproduction in any medium or format, as long as you give appropriate credit to the original author(s) and the source, provide a link to the Creative Commons licence, and indicate if changes were made. The images or other third party material in this article are included in the article’s Creative Commons licence, unless indicated otherwise in a credit line to the material. If material is not included in the article’s Creative Commons licence and your intended use is not permitted by statutory regulation or exceeds the permitted use, you will need to obtain permission directly from the copyright holder. To view a copy of this licence, visit http://creativecommons.org/licenses/by/4.0.

The original article has been corrected.

