# Supplementary material for: Assessment of renal congestion in a rat model with congestive heart failure using superb microvascular imaging
Source: J Med Ultrason (2001). 2024 Jan 11;51(2):159–68. doi: 10.1007/s10396-023-01396-7 (PMC11928411; doi:10.1007/s10396-023-01396-7)
Supplement: Supplementary file 1 — Supplementary file1 (DOCX 9044 KB) [file 10396_2023_1396_MOESM1_ESM.docx]

**Supplementary manuscript**

**Supplementary figure1: Renal edematous area**

These panels show pathological images of Masson's trichrome-stained kidneys. The left panel is the control group and the right panel is the HF group.

*HF* heart failure
